# HPV vaccination in Iran: expert perspectives on family dynamics and barriers

**DOI:** 10.1186/s12905-026-04269-6

**Published:** 2026-01-30

**Authors:** Monireh Faghir-Ganji, Pouria Feizabadi, Alireza Ansari-Moghaddam, Narjes Abdolmohammadi, Babak Eshrati

**Affiliations:** 1 Student Research Committee, Iran University of Medical Sciences, Tehran, Iran; 2 Department of Epidemiology, School of Public Health, Iran University of Medical Sciences, Tehran, Iran; 3 Health Promotion Research Center, Zahedan University of Medical Sciences, Zahedan, Iran; 4 Department of Epidemiology, School of Public Health, Tehran University of Medical Sciences, Tehran, Iran; 5 Preventive Medicine and Public Health Research Center, School of Medicine, University of Medical Sciences, Tehran, Iran

**Keywords:** Human papillomavirus, Human papillomavirus vaccination, Qualitative study, Expert perspectives, Family dynamics, Barriers, Iran.

## Abstract

**Background and aim:**

Cervical cancer, primarily caused by Human Papillomavirus, remains a significant global health concern. Despite vaccine availability, uptake rates are low in many conservative settings, including Iran, where context-specific factors influencing vaccination are often overlooked. This qualitative study aimed to explore and articulate the perceptions, experiences, and actionable recommendations of health and social experts concerning the barriers, facilitators, and the perceived family implications of Human Papillomavirus vaccination in Iran.

**Methods:**

This qualitative inquiry employed Conventional Content Analysis. Using Maximum Variation Purposive Sampling, 18 female experts and professionals (Obstetricians/Gynecologists, health educators, laboratory staff, and academic researchers) were recruited from major medical universities in Tehran (January 2025 to September 2025). Data were gathered through five Focus Group Discussions (FGDs) and seven semi-structured In-depth Individual Interviews. Methodological rigor was ensured via techniques like Member Checking. Data were analyzed using MAXQDA 2020 software.

**Results:**

Participants included 18 female experts (mean age 44 ± 10.7 years). Thematic analysis identified 13 sub-themes categorized into five domains: (1) family awareness and information sources; (2) family attitudes and beliefs; (3) family decision-making; (4) systemic and economic barriers; and (5) family wellbeing and policy. A central finding was the synergistic interplay between high economic costs and deep-seated social stigma. This linkage perpetuates low awareness and fuels moral misconceptions (e.g., vaccine as a “moral license”), which often lead to severe marital conflicts, infidelity accusations, and psychosocial distress within the family unit.

**Conclusion:**

From the Iranian experts’ perspective, vaccination uptake is primarily hindered by high cost, limited access, and deeply rooted social stigma associated with sexually transmitted infections. Key actionable recommendations include full insurance coverage, introducing affordable domestic vaccines, and implementing culturally tailored public health education to address moral concerns. These findings provide crucial evidence for policymakers to design targeted interventions that improve Human Papillomavirus vaccine coverage and protect family health in Iran.

## Introduction

Cervical cancer remains a significant global health issue, with the GLOBOCAN report indicating that 604,127 women were diagnosed and 341,831 women died from this disease worldwide [1, 2]. It ranks as the fourth most common cancer among women, following breast, colorectal, and lung cancers, and is primarily caused by Human Papillomavirus infection. Over 200 types of Human Papillomavirus have been identified, and numerous studies have established a link between Human Papillomavirus infections and various cancers, including oral, pharyngeal, vaginal, vulvar, penile, and anal cancers. According to the Centers for Disease Control and Prevention, Human Papillomavirus contributes to more than 21,000 cancer cases in women and approximately 15,000 cases in men annually in the United States [3]. The high prevalence of Human Papillomavirus-related cancers imposes a significant economic and public health burden on society.

Global efforts to enhance Human Papillomavirus vaccination are vital for combating cervical cancer. The Human Papillomavirus vaccine was first approved in the United States in 2006. Despite its availability, vaccination rates among adults remain significantly lower than those among adolescents, with notable gender and racial disparities [4]. Global coverage for the first dose of the Human Papillomavirus vaccine among girls reached 27% by 2023, largely attributed to the implementation of the World Health Organization’s Global Strategy aimed at eliminating cervical cancer by 2030 [5, 6]. This strategy includes ambitious targets such as vaccinating 90% of girls by age 15 [5, 6]. More than 140 countries have integrated the Human Papillomavirus vaccine into their national immunization programs [7]. Despite these advancements, significant disparities persist globally, with low- and middle-income countries struggling with lower vaccination coverage despite facing a greater cervical cancer burden [8, 9]. Furthermore, lack of awareness among eligible adults contributes to non-vaccination, with studies indicating that many are uninformed about Human Papillomavirus vaccination, and men may mistakenly believe they are not at risk [10, 11].

Currently, six licensed Human Papillomavirus vaccines are available globally, including bivalent, quadrivalent, and nonavalent versions. These vaccines provide robust protection against specific Human Papillomavirus strains, particularly types 16 and 18, which are responsible for approximately 70% of cervical cancer cases worldwide [12]. In Iran, the Human Papillomavirus vaccine is primarily available through the private sector, often at a high cost and without broad national coverage. This context is further complicated by profound cultural and religious beliefs surrounding sexuality, the social stigma associated with sexually transmitted infections, and a general lack of awareness about Human Papillomavirus [13–15]. These unique contextual factors create substantial systemic, economic, and socio-cultural barriers to vaccination uptake.

The existing literature on Human Papillomavirus vaccination in Iran and similar conservative settings has predominantly focused on quantitative assessments of knowledge, attitudes, and prevalence. There is a critical knowledge gap concerning the in-depth, nuanced perceptions of key stakeholders (experts and professionals) regarding systemic barriers, emotional consequences, and the resulting influence on family decision-making and well-being [13–15]. The logical link between Human Papillomavirus vaccination and family dynamics (i.e., parental decision-making, marital conflict over perceived risk, and the emotional burden of stigma) is a central mechanism that needs conceptual grounding, particularly from the perspective of those who counsel the public and implement policy.

The existing literature on Human Papillomavirus vaccination in Iran and similar conservative settings has predominantly focused on quantitative assessments of knowledge, attitudes, and prevalence among the general public or students. However, a significant research gap remains regarding how systemic failures, such as high costs and lack of a national program, interact with deeply rooted socio-cultural stigmas to disrupt family dynamics. Specifically, current evidence fails to explain the professional-level interpretation of how these barriers lead to marital conflicts, parental decision-making paralysis, and the ‘supportive paradox’ within Iranian families. There is a lack of qualitative insight from the perspective of health and social experts who bridge the gap between policy and family-level implementation. Understanding these nuanced mechanisms is essential for developing interventions that address not just individual knowledge, but the family-centered emotional and social burden of the vaccine.

Therefore, this qualitative study aims to explore and articulate the perceptions, experiences, and actionable recommendations of health and social experts and professionals concerning the barriers, facilitators, and the perceived family implications of Human Papillomavirus vaccination within the Iranian context.

## Methods

### Study design and rationale

This qualitative research was conducted using a Conventional Content Analysis approach. This approach was highly suitable because it allowed categories and themes to be derived directly from the textual data, without imposing pre-determined theories or frameworks [16, 17].

This inductive method was essential to uncover the complex, context-specific perspectives of experts in Iran, thereby generating new knowledge and valid insights into the multifaceted challenges of Human Papillomavirus vaccination. The research was conducted in January 2025 to September 2025.

### Participants and sampling

The study population consisted of 18 female experts and professionals involved in health, research, or education affiliated with two major medical universities in Tehran, Iran. Participants included Obstetricians/Gynecologists, health educators, laboratory staff, and academic researchers. The term ‘experts and professionals’ was broadly defined to include individuals with direct practical or academic experience concerning HPV, women’s health, or public health policy, encompassing specialized roles such as clinical staff, academic researchers, and advanced graduate students involved in related research.

### Sampling strategy and saturation

To ensure the Transferability of the findings, Maximum Variation Purposive Sampling was employed. This strategic approach was chosen to recruit participants with diverse perspectives based on key characteristics, including workplace (clinical vs. academic), work experience, job title, and educational level.

*Participant recruitment process*: Initial potential participants were first identified through professional networks and formal referrals from department heads within the selected medical universities in Tehran. Following this identification, the first author provided a detailed explanation of the study’s purpose and obtained written informed consent from all participants prior to their inclusion in the study.

Inclusion criteria required participants to be female experts or professionals with direct involvement or knowledge of Human Papillomavirus, women’s health, public health policy, or family dynamics within the Iranian health system.

Exclusion criteria included: (1) any inability or unwillingness to provide written informed consent for participation, or (2) individuals who were not female experts or professionals with direct involvement or knowledge of the specified thematic areas.

Participant recruitment and data collection continued iteratively for approximately 9 months until Data Saturation was achieved. The Sample Size was determined solely by the principle of Data Saturation. Data analysis (Open Coding and Creating Categories) was performed concurrently with data collection. Saturation was confirmed when no new codes, concepts, or substantive insights emerged from subsequent interviews, indicating that the data collected was sufficient to provide a comprehensive and nuanced understanding of the phenomenon from the experts’ viewpoint.

### Data collection methods

Data were gathered through a combination of five Focus Group Discussions (FGDs) and seven semi-structured In-depth Individual Interviews. The interviews were conducted using a semi-structured guide to ensure all relevant topics were covered while allowing for in-depth exploration. Sample questions included: (1) *“In your professional opinion*,* what are the primary cultural and systemic barriers to HPV vaccination in Iran?”* (2) *“How do family dynamics and parental beliefs influence the decision-making process for adolescent vaccination?”* (3) *“Based on your experience*,* what are the emotional and marital consequences for families when dealing with an HPV-related diagnosis?”* Data collection through both individual interviews and Focus Group Discussions (FGDs) continued until data saturation was achieved. The process of detecting saturation was systematic; the research team conducted ongoing data analysis alongside data collection. Saturation was confirmed when the final three individual interviews and the last focus group session yielded no new codes, categories, or conceptual insights, indicating that the data had reached a point of redundancy and the thematic framework was comprehensive.

### Interview tool development and validation

Each interview utilized a semi-structured interview guide. The guide was developed based on a thorough review of the literature and consultation with an expert panel, and was pilot-tested. The key domains covered included: awareness of the Human Papillomavirus vaccine, challenges related to sexually transmitted infections stigma, impact on family and marital relationships, and policy suggestions.

### Procedure and Language

Interviews and discussions were conducted in a private, non-judgmental setting in Tehran in January 2025 to September 2025. All sessions were recorded with participant permission and immediately transcribed verbatim by the first author (MFG). The language used for data collection and transcription was Persian.

### Reflexivity and researcher’s role

The first author (MFG), a female PhD student and member of the research team, conducted all semi-structured interviews. While the participants were primarily affiliated with the two medical universities in Tehran, institutions with which the interviewer also held a professional affiliation, the interviewer had no prior professional or personal relationship with any participant before the study. This deliberate separation helped to ensure a balanced approach and minimize pre-existing biases associated with hierarchical or social roles.

To facilitate participants’ comfortable and honest expression, and to actively manage potential power dynamics inherent in the research setting, the interviewer adopted a non-judgmental stance and explicitly stated that “there are no right or wrong answers” during the introductory phase of each interview.

Regarding reflexive practices, the interviewer maintained a detailed field journal to document initial assumptions, subjective feelings, and contextual observations arising during data collection. Regular team debriefings and reflexive discussions were also held with the corresponding author (BE) to systematically examine the interviewer’s influence and assumptions. This process of active bracketing of biases throughout the data collection and analysis phase was crucial for enhancing the Confirmability and Trustworthiness of the findings.

### Data analysis

Data analysis was performed using Conventional Content Analysis, leveraging MAXQDA 2020 software. This inductive approach was essential as it allowed categories and themes to be derived directly from the textual data, minimizing the imposition of pre-existing theoretical perspectives. The organization of the qualitative data followed three main steps: Open Coding, Creating Categories, and Abstraction into the final main thematic domains.

### Trustworthiness and rigor

Rigour was established using standard qualitative criteria:

- Credibility: Ensured via Member Checking and interviewer neutrality.
- Dependability and Confirmability: Ensured via Independent Analysis of a random sample of transcripts by a second team member (AAM). Any discrepancies were resolved through critical discussion and consensus with the corresponding author (BE).
- Transferability: Ensured via Maximum Variation Sampling and Thick Description of the context and participant characteristics.

## Results

### Participant characteristics

The study was conducted between January and September 2025, involving 18 female experts and professionals affiliated with the Iran University of Medical Sciences and Tehran University of Medical Sciences. The participants’ demographic details, including age and education levels, are summarized in Table 1. The mean age of the participants was 44 ± 10.7 years, with professional experience ranging from 2 to 32 years.

**Table 1:** Demographic characteristics of participants based on basic information (*N* = 18)

Interview number	Age (in year)	Marital status	Interview duration (minutes)	Work experience (years)	Job	Education
1	55	Married	22	23	Obstetricians	Expert
2	46	Married	86	19	Academic staff of Iran University of Medical Sciences	PhD
3	47	Married	86	23	Academic staff of Iran University of Medical Sciences	PhD
4	40	Married	35	12	Academic staff of Tehran University of Medical Sciences	PhD
5	52	Married	55	15	Laboratory Specialist	PhD
6	57	Married	45	16	Obstetricians	Expert
7	32	Single	21	4	Surgical specialty	Student
8	37	Single	21	4	Surgical specialty	Student
9	33	Single	21	4	Surgical specialty	Student
10	41	Married	15	10	Obstetricians	Expert
11	44	Married	63	5	PhD in Epidemiology	PhD
12	38	Married	63	2	PhD in nanotechnology	PhD
13	63	Married	40	32	Specialist in breast surgery	Expert
14	43	Married	40	16	Research staff	Master
15	33	Single	20	2	Master of Professional Health	Student
16	27	Married	32	3	Master of Nursing	Master
17	39	Married	32	15	Academic staff of Iran University of Medical Sciences	PhD
18	65	Married	15	32	Obstetricians	Expert

### Data collection process

Information was gathered through seven semi-structured In-depth Individual Interviews (IDIs) and five Focus Group Discussions (FGDs). The specific characteristics of these data collection sessions, such as duration and participant distribution for each session, are detailed in Table 3.

**Table 3:** Technical characteristics of interviews and focus group discussions

Row	Number of Participants	Occupation	Interview Duration (Minutes)	Data Collection Method
1	2	Nurses	86	Focus Group Discussion (FGD 1)
2	2	Nurses	32	Focus Group Discussion (FGD 2)
3	2	Epidemiologist and Nanotechnology Specialist	63	Focus Group Discussion (FGD 3)
4	3	Surgery Student	31	Focus Group Discussion (FGD 4)
5	2	Breast Surgery Specialist	40	Focus Group Discussion (FGD 5)
6	1	Gynecologist	22	In-Depth Interview 1
7	1	Gynecologist	15	In-Depth Interview 2
8	1	Gynecologist	15	In-Depth Interview 3
9	1	Virology Specialist	35	In-Depth Interview 4
10	1	Gynecologist	45	In-Depth Interview 5
11	1	Laboratory Specialist	55	In-Depth Interview 6
12	1	Graduate Student	20	In-Depth Interview 7
Total	18		454	5 FGDs and 7 Individual Interviews

### Thematic framework and domains

The systematic analysis of the qualitative data led to the identification of 13 sub-themes, which were organized into five primary thematic domains. An integrated synthesis of these domains, including sub-themes, frequency of mentions, and representative quotes, is presented in Table 2.

**Table 2:** Integrated thematic analysis of HPV vaccination barriers and family dynamics

Category	Themes	Key Illustrative Quotes	Frequency (*N* = 18)
**1. Family Awareness & Info Sources**	♣ Family awareness and knowledge level	“Relying on non-standard internet sources causes misunderstanding and unnecessary fear.” (IDI 1)	16
	♣ Family information sources		
	♣ Family understanding of the importance of vaccination		
**2. Family Attitudes & Beliefs**	♣ Positive and negative beliefs and attitudes towards the vaccine	“Families worry the vaccine grants ‘permission’ for risky sexual behavior.” (FGD 2)	15
	♣ Family perception of the risks and benefits of the vaccine		
**3. Family Decision-Making**	♣ The impact of vaccination on communication and interaction between family members	“STI-related shame can lead to severe family tensions, emotional divorce, or even violence.” (IDI 4)	14
	♣ The role of parents in decision-making about vaccination		
**4. Systemic & Economic Barriers**	♣ Access and availability of the vaccine	“High prices create a divide; some patients prefer surgery over expensive prevention.” (IDI 6)	17
	♣ Costs related to vaccination		
	♣ Family trust in the healthcare system		
	♣ Social and cultural attitudes		
**5. Family Wellbeing & Policy**	♣ The role of officials and policymakers in promoting vaccination	“Education in high schools is the best solution, but it requires a shift in policymakers’ mindsets.” (FGD 3)	13
	♣ Impact of vaccination on the mental health and well-being of families		

The five identified domains are:

1. Family Awareness and Info Sources
2. Family Attitudes and Beliefs
3. Family Decision-Making
4. Systemic and Economic Barriers
5. Family Wellbeing and Policy

### Categorization and thematic domains

1. Family Awareness & Info Sources.
  The participants emphasized that improving public awareness, especially among vulnerable groups, is a core strategy for increasing vaccine uptake.
  Family awareness and knowledge level: Awareness remains insufficient among lower socio-economic classes. A significant gap exists in linking infection symptoms to the vaccine’s preventive role. One expert noted a pregnant patient with advanced warts who *“had no idea… she just didn’t connect it to the vaccine at all”* (IDI 5).
  Family information sources: Reliance on non-standard internet sources and social media (e.g., Instagram) causes fear-based misunderstandings. As noted in the results, *“Relying on non-standard internet sources causes misunderstanding and unnecessary fear”* (IDI 1).
  Family understanding of the importance of vaccination: Stigma limits the perceived necessity of the vaccine to “high-risk” groups only, deterring average families from seeking immunization (FGD 5, IDI 5).
2. Family Attitudes and Beliefs.
  Family attitudes are characterized by a paradox of medical fear and moral concerns.
  Positive and negative beliefs and attitudes towards the vaccine: While there is awareness of the virus’s dangers, there is deep-seated distrust toward new vaccines. Experts highlighted that *“Patients are terrified [of cancer]… and that fear comes from them not really knowing the vaccine’s benefits”* (IDI 4).
  Family perception of the risks and benefits of the vaccine: A primary barrier is the belief that vaccination encourages early sexual activity. As captured in the consensus: *“Families worry the vaccine grants ‘permission’ for risky sexual behavior”* (FGD 2).
3. Family Decision-Making.
  The dynamics of family interaction significantly influence the choice to vaccinate, often hampered by social sensitivities.
  The impact of vaccination on communication and interaction between family members: Experts noted that the shame associated with HPV often leads to secrecy and severe domestic tensions. As highlighted in the findings, *“STI-related shame can lead to severe family tensions*,* emotional divorce*,* or even violence”* (IDI 4). Vaccination is seen as a tool to mitigate these social harms.
  The role of parents in decision-making about vaccination: Parents are the primary decision-makers but are often paralyzed by the fear that vaccination might encourage high-risk behaviors. This “assumption of innocence” prevents proactive health measures for their children (FGD 1).
4. Systemic & Economic Barriers.
  Structural challenges and financial constraints form a significant wall against widespread immunization.
  Access and availability of the vaccine: Limited supply in pharmacies and the difficulty of tracking foreign vaccines create a high barrier for families. Many report long wait times or having to use informal networks to find doses (FGD 1, FGD 2).
  Costs related to vaccination: The high price of foreign vaccines often forces families to choose “treatment over prevention.” As noted, *“High prices create a divide; some patients prefer surgery over expensive prevention”* (IDI 6).
  Family trust in the healthcare system: There is a notable deficit of trust, especially toward domestic vaccines, fueled by a lack of national education. Families often demand more proof of safety before uptake (FGD 2).
  Social and cultural attitudes: The pervasive stigma surrounding HPV is a major deterrent, with experts explicitly stating that the social burden of this infection is often perceived as *“worse than HIV”* (IDI 7).
5. Family Wellbeing & Policy.
  Addressing vaccination requires a shift from individual responsibility to systemic policy changes.
  The role of officials and policymakers in promoting vaccination: Participants called for a “cultural shift” at the governmental level, suggesting that vaccination be integrated into routine national programs and school curricula. One participant noted: *“Education in high schools is the best solution*,* but it requires a shift in policymakers’ mindsets”* (FGD 3).
  Impact of vaccination on the mental health and well-being of families: The psychological stress of potential infidelity accusations remains a core challenge. Vaccination is recommended not just as a medical intervention, but as a way to protect the marital stability and mental health of families (IDI 2, FGD 1).

## Discussion

This study utilized expert perspectives to examine the barriers to acceptance of Human Papillomavirus (HPV) vaccination and its perceived influence on family dynamics in Iran. The findings were consolidated into five main thematic domains: Family Awareness, Attitudes and Beliefs, Influence on Family Decision-Making, Systemic and Economic Impediments, and Impact on Family Mental Health.

The innovative contribution of this research is the qualitative interpretation of the systematic link between economic barriers and deeply rooted social stigma. In the Eastern Mediterranean Region (EMRO), including Iran, lack of knowledge and economic constraints have been identified as major barriers [18]. However, our study suggests that in Iran, these factors do not act in isolation; rather, they form a synergistic barrier that renders prevention inaccessible for the majority of the population.

### Economic Barriers to HPV Vaccination

As emphasized by the participating experts, enhancing public awareness through extensive education is a pivotal strategy for increasing vaccine uptake [19]. However, awareness remains notably insufficient among vulnerable socio-economic groups. This knowledge deficit aligns with reports from other Muslim-majority countries, where religious and cultural sensitivities frequently impede open discourse on sexual health [20].

These educational gaps are significantly exacerbated by formidable economic barriers. The prohibitive cost of imported vaccines (e.g., Gardasil), coupled with a lack of comprehensive insurance coverage, renders immunization unaffordable for a large portion of the Iranian population. Previous economic evaluations indicate that at current market prices, HPV vaccination is not cost-effective for the national health system [21]. Specifically, research suggests that vaccine prices would need to decrease by 82–85% to meet the threshold for a cost-effective public health measure in Iran [15, 21].

Unlike international initiatives such as Gavi, which subsidize vaccine costs for low-income nations, the financial burden in Iran falls directly on families, often forcing a shift in priority from “prevention to treatment”. This situation is further evidenced by the high mortality-to-incidence ratio of cervical cancer in Iran, suggesting that diagnoses often occur at advanced stages due to suboptimal prevention and screening infrastructure [22]. While the introduction of locally produced vaccines may offer a more affordable alternative and alter future cost-effectiveness calculations, current uptake is still hindered by a lingering distrust of domestic products and periodic pharmacy shortages [22–24].

### Social Stigma: Goffman’s Theory and Honor Culture

A unique and critical finding is that HPV-related stigma in Iran is often perceived as more severe than HIV-related stigma. This can be interpreted through Goffman’s theory of social stigma, where the infection is viewed as a “spoiled identity” that threatens the individual’s moral standing [25, 26]. In the context of Iran’s “Honor Culture,” where family reputation and sexual “purity” are paramount, HPV is not seen merely as a medical issue but as a moral failure [27, 28]. While HIV is often associated with clinical or broader social risks, HPV is specifically tied to the fear of “licensing” premarital sexual behavior. This fear leads parents to perceive the vaccine as a catalyst for risky behavior rather than a life-saving intervention. Our study demonstrates how this stigma leads to secrecy, marital conflict, and even the risk of “infidelity accusations,” highlighting the urgent need to separate the “issue of treatment from ethics“ [29–31].

#### Specific policy recommendations

To address these multi-layered barriers, specific and multi-sectoral policy interventions are required:

- *Media and Cultural De-stigmatization*: The Ministry of Health should collaborate with the media to integrate health messaging into popular TV dramas and series. By portraying vaccination as a tool for “family stability” rather than a response to “risky behavior,” the stigma can be reduced.
- *Engagement of Religious Scholars*: Involving clerics and religious scholars in health messaging is essential. Their endorsement can reframe vaccination as a moral duty to protect health and family life, bridging the gap between traditional values and modern medicine.
- *Targeted Financial Support*: The government must implement targeted financial support packages and full insurance coverage for low-income families to eliminate the class divide in vaccine access.
- *School-Based Programs*: Normalizing the vaccine through mandatory school-based programs can remove the burden of individual decision-making from parents and reduce the moral weight of the vaccine.

## Conclusion

In conclusion, this study demonstrates that HPV vaccine uptake in Iran is hindered by a synergistic mechanism where high economic cost intersects with deep social stigma. The primary barriers are the unaffordable price of imported vaccines and a critical lack of insurance coverage. Crucially, this economic deficit is exacerbated by a pervasive socio-cultural stigma, rooted in the “Honor Culture”, that fuels parental fear and psychosocial distress. To address this challenge, we recommend a two-pronged approach: immediate financial measures (full insurance and trusted domestic supply) and culturally tailored interventions (public education in schools and media). Moving from a descriptive approach to a structured policy shift is essential to protect the health and well-being of Iranian families.

### Strengths and limitations

#### Strengths and innovative contributions

This study makes a substantial and innovative contribution to the literature on Human Papillomavirus vaccination, leveraging a deep qualitative methodology and Conventional Content Analysis to fill a critical knowledge gap left by prior quantitative research in Iran. The foremost strength is the unveiling of a strong systemic link between various barriers in the conservative cultural context: demonstrating how high cost and limited vaccine access appear to contribute to low public awareness, negative beliefs, and the ultimate escalation of psychosocial harm within families. By utilizing expert perspectives (health and social professionals), the research yielded specific insights into the observed consequences of the virus on family dynamics (including the stigma of infidelity, shame, and marital conflict), framing the issue as a “complex socio-familial challenge.” Furthermore, the study produced highly practical and culturally relevant recommendations for policymakers in areas such as insurance coverage and targeted public health education. Finally, methodological rigor was ensured through techniques like Maximum Variation Purposive Sampling (to guarantee diverse views) and Member Checking (to confirm findings accuracy).

### Limitations and recommendations for future research

Despite its strengths, this research has limitations that should be acknowledged. First, the study’s exclusive focus on female experts from two universities in Tehran may introduce both gender and geographical bias. While the choice of female participants was methodologically conscious, given their central role as primary health decision-makers and counselors in the Iranian domestic sphere, it inadvertently ignored the perspectives of male professionals and fathers/husbands, whose influence on family dynamics is equally significant.

Additionally, the research focused on expert viewpoints rather than the direct experiences of the target population (such as vaccinated individuals or those infected), which may limit the applicability of the findings to broader community-level behaviors. Consequently, we recommend that future research adopt a more diverse sampling strategy, including male participants and participants from different geographical regions of Iran. Furthermore, investigating the direct experiences of those affected by STIs will provide a more comprehensive understanding of the socio-cultural barriers and facilitators, ultimately aiding the design of more robust, gender-inclusive interventional studies.

## Data Availability

The qualitative datasets (interview transcripts) analyzed during the current study are not publicly available due to the need to maintain the confidentiality and anonymity of the participating staff, but are available from the corresponding author on reasonable request.

## Abbreviations

HPV: Human Papillomavirus
STI: Sexually Transmitted Infection
LMICs: Low- and Middle-Income Countries
FGD: Focus Group Discussion
IDI: In-depth Individual Interview
CDC: Centers for Disease Control and Prevention
GAVI: Global Alliance for Vaccines and Immunization
WHO: World Health Organization
GLOBOCAN: Global Cancer Observatory
EMRO: Eastern Mediterranean Region
